# Supra‐Blan_2_t score as a multisystem‐based risk score to predict poor 3‐month outcome in acute ischemic stroke patients with intravenous thrombolysis

**DOI:** 10.1111/cns.14381

**Published:** 2023-07-30

**Authors:** Huijuan Jin, Qiwei Peng, Min Li, Shuai Sun, Jinghua Zhou, Jichuan Hu, Ming Huang, Xinglong Chen, Yanan Li, Yifan Zhou, Yan Wan, Candong Hong, Shengcai Chen, Bo Hu

**Affiliations:** ^1^ Department of Neurology, Union Hospital, Tongji Medical College Huazhong University of Science and Technology Wuhan China; ^2^ Department of Neurology The Second People's Hospital of China Three Gorges University Yichang China; ^3^ Department of Neurology The First Clinical Medical College of China Three Gorges University Yichang China; ^4^ Department of Neurology People's Hospital of Dongxihu District Wuhan China; ^5^ Department of Neurology Hubei Provincial Hospital of Integrated Chinese and Western Medicine Wuhan China

**Keywords:** acute ischemic stroke, outcome, prediction score, remote organ, thrombolysis

## Abstract

**Aim:**

To develop and validate a novel weighted score integrating multisystem laboratory and clinical variables to predict poor 3‐month outcome (mRS score of 3–6) in acute ischemic stroke (AIS) patients with intravenous thrombolysis (IVT) therapy.

**Methods:**

We retrospectively analyzed data from Trial of Revascularization Treatment for Acute Ischemic Stroke study. The Supra‐Blan_2_t score was derived using the data on age, the National Institutes of Health Stroke Scale score, history of atrial fibrillation, blood sugar level, neutrophil count, direct bilirubin level, platelet–lymphocyte ratio, and TnI level in the derivation cohort of 433 patients, and validated in a cohort of 525 patients. Furthermore, we compared the performance of the Supra‐Blan_2_t score with DRAGON, TURN, and SPAN‐100 scores.

**Results:**

The discrimination capacity in the derivation and validation cohorts was good for poor 3‐month outcome (the area under the curve was 0.821 and 0.843, respectively). The cumulative incidence of poor 3‐month outcome significantly increased across risk categories in the derivation (low‐risk, 9.2%; medium‐risk, 17.4%; and high‐risk, 58.8%) and validation cohorts (12.7%, 36.5%, and 73.6%, respectively). The performance of the Supra‐Blan_2_t score was similar to or superior to DRAGON, TURN, and SPAN‐100 scores.

**Conclusion:**

The Supra‐Blan_2_t score, based on easily available multisystem laboratory and clinical variables, reliably predicted poor 3‐month functional outcome in AIS patients treated with IVT therapy featuring good calibration and discrimination.

## Graphical abstract

Multisystem parameters and clinical variables involvement in Supra‐Blan_2_t prediction score. Multisystem included cardiovascular system, endocrine system, immune system, and digestive system, while clinical variables included age, admission National Institutes of Health Stroke Scale score, and history of atrial fibrillation.

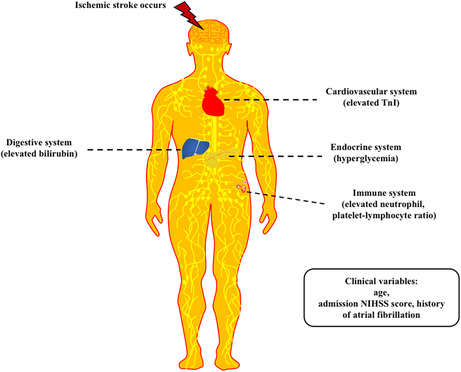

## 1 INTRODUCTION

Intravenous thrombolysis (IVT) with tissue‐type plasminogen activator within the therapeutic time window of 4.5 h has been the only approved effective pharmacological reperfusion treatment for patients with acute ischemic stroke (AIS).^1^ Despite its vast benefits, IVT is not equally effective in all patients, nor is it without significant risks. It was reported that about half of patients receiving thrombolysis therapy tended to have poor long‐term functional outcomes.^2^, ^3^ Early identification of patients unlikely to reap long‐term benefits from IVT is important to both neurologists and patients. On the one hand, it contributes to rapid arrangements for invasive add‐on therapeutic strategies, such as endovascular treatment. On the other hand, early estimation of prognosis may help determine the clinical cost‐effectiveness, length of hospital stay, and rehabilitation therapy.

As a systemic disease, ischemic stroke usually induces detrimental effects on multiple remote organ systems, mainly, but not limited to, the cardiovascular, immune, and digestive systems, which play an essential role in the disability and mortality in patients with AIS.^4^ Whether the long‐term functional outcome in patients with IVT was also affected by the aforementioned remote organ abnormalities is not yet clear. To date, several predictive models^5^, ^6^, ^7^, ^8^, ^9^ were developed to create prognostication scores for patients with IVT, but they did not systematically evaluate the role of widespread end‐organ dysfunction. Few models have been widely accepted and applied clinically due to various individual and interacting factors. Clinicians still face challenges in translating these predictive models into daily practice.

In view of the gaps in practical applications of aforementioned models, this study aimed to develop weighted risk scores based on simple multisystem parameters and clinical variables to predict poor 3‐month functional outcome in the derivation cohort in a multicenter clinical Trial of Revascularization Treatment for Acute Ischemic Stroke (TRAIS) study. This study further involved an external validation on another group of patients in a TRAIS study to validate the accuracy of the score. Furthermore, its performance was also compared with that of other three recognized scoring systems, including DRAGON, TURN, and SPAN‐100.

## 2 MATERIALS AND METHODS

The data of this study were derived from the TRAIS study performed in two nonoverlapping derivation‐validation cohorts between January 2018 and January 2022. The TRAIS study was a multicenter prospective‐cohort study of consecutive patients receiving IVT treatment within time window after the onset of symptoms admitted to 14 hospitals in the Hubei province. We enrolled patients applying the following inclusion criteria: (1) aged ≥18 years; (2) diagnosed with stroke; (3) cerebral hemorrhage ruled out using cerebral computed tomography (CT) scan; and (4) no contraindication for thrombolysis therapy.

**FIGURE 1:**
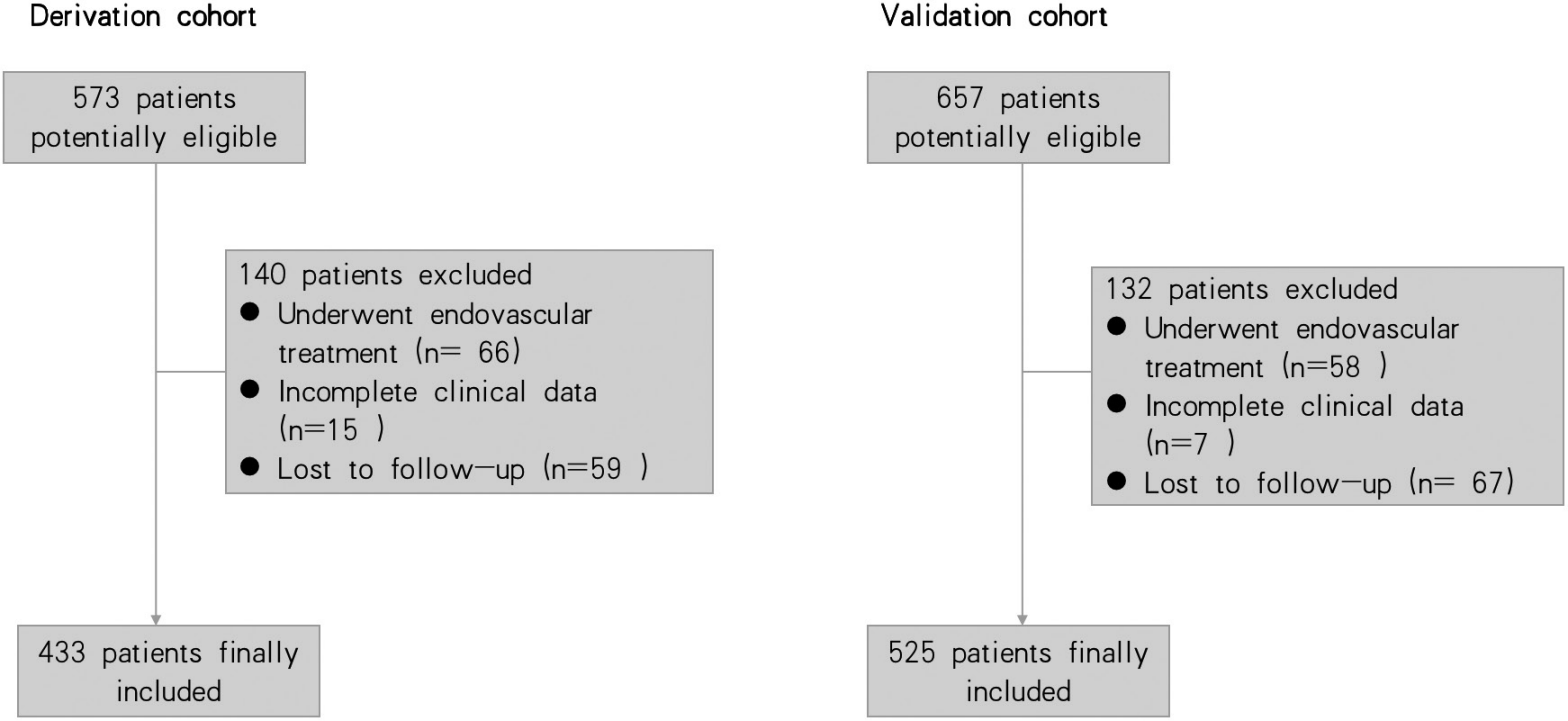
The flowchart of study population.

A total of 573 and 657 patients were eligible for inclusion in the derivation and validation cohorts, respectively. After screening, 433 and 525 patients were finally included in each of the two cohorts, respectively (Figure 1). The derivation cohort comprised 433 patients with AIS receiving IVT therapy (0.9 mg/kg body weight) at the Wuhan Union Hospital. The validation cohort comprised 525 patients with AIS receiving the same dose of IVT therapy at the Wuhan Union Hospital West Campus, the Wuhan Union Hospital Jinyinhu Campus, the People's Hospital of Dongxihu District, the Hubei Provincial Hospital of Integrated Chinese and Western Medicine, the Central People's Hospital of Yichang, the First People's Hospital of Yichang, the Second People's Hospital of Yichang, the Central Hospital of Hefeng County, the People's Hospital of Honghu, the People's Hospital of Jingshan, and the First People's Hospital of Jiangxia District, Wuhan Red Cross Hospital, Puren Hospital of Wuhan. All participants provided written informed consent upon inclusion in this study, which was in accordance with the ethical standards stated in the 1975 Declaration of Helsinki. The study protocol was approved by the ethics committee of the Union Hospital, Tongji Medical College, Huazhong University of Science and Technology, Wuhan, China (ChiCTR2000033456).

The time window for IVT is extended up to 9 h guided by perfusion imaging technology.^10^ The peripheral venous blood samples from patients were collected prior to thrombolysis therapy upon admission, and the demographic characteristics, clinical features, and medical history were also obtained from patients. The severity of the stroke was assessed by experienced neurologists using the National Institutes of Health Stroke Scale (NIHSS). Patients with blood pressure ≥140/90 mmHg or self‐reported physician‐diagnosed hypertension or current use of antihypertensive medication were identified as hypertension.^11^ Patients with abnormal blood lipid levels (total cholesterol >6.22 mmol/L; triglyceride >2.26 mmol/L; and low‐density lipoprotein cholesterol >4.14 mmol/L) or self‐reported history of physician‐diagnosed hyperlipidemia were considered as hyperlipidemic.^12^ Diabetes mellitus (DM) was identified in patients with fasting glucose levels >7.0 mmol/L or those taking oral hypoglycemic drugs or insulin or those self‐reporting physician‐diagnosed DM.^11^ Atrial fibrillation (AF) was defined as a medical history of persistent or paroxysmal AF, or as confirmed by at least one electrocardiogram or the presence of AF during hospitalization.^13^

In this study, we focused on the modified Rankin Scale (mRS) score after 3 months, which was used to assess the functional outcome of patients by experienced neurologists who were blinded for treatment allocations to patients. Good functional outcome was observed in patients with an mRS score of 0–2, while poor outcomes were reported in patients with an mRS score of 3–6.

### 2.1 Statistical analysis

The Kolmogorov–Smirnov test of normality was used to assess data distribution. The continuous and categorical variables of patients' baseline characteristics in each cohort were presented as mean ± standard deviation (SD) or median (interquartile range) and numbers (percentages), respectively. The differences in the baseline characteristics of each cohort were tested using chi‐square tests for categorical characteristics and analysis of variance for continuous characteristics. Multivariable logistic regression was used to calculate odds ratios (ORs) and 95% confidence intervals (CIs). Variables with univariate *p* < 0.05 were used for multivariate logistic regression analysis to identify multisystem and clinical variables associated with the clinical outcome in the derivation cohort. Variables were categorized into continuous and ordinal variables for clinical applications, and the cutoff values were chosen based on Youden's index.^14^ C statistic, which is equivalent to the area under the curve (AUC) on the receiver‐operating characteristic (ROC) curve, was used to estimate the discriminative power of prediction score for clinical outcome, and the Hosmer–Lemeshow test was used to evaluate the goodness‐of‐fit.

The weighted risk score for predicting poor 3‐month outcome was derived from the point estimate of each variable using the *β* coefficients in the derivation cohort. Relative point values were assigned by dividing each regression coefficient by half of the minimum coefficient and rounding it to the nearest integer (the parameter <1 was assigned a score of 1). The diagnostic accuracy analysis was performed using sensitivity, specificity, positive predictive values (PPVs), negative predictive values (NPVs), and likelihood ratios based on different risk cutoff scores.

In the derivation cohort, we investigated the relationship of the score with the risk of poor 3‐month outcome by analyzing the distribution of poor 3‐month outcome across increasing levels of the score. The multivariable logistic regression analysis was performed using the data on age, history of AF, admission NIHSS score, admission blood sugar level, admission neutrophil count, admission direct bilirubin level, admission TnI level, and platelet–lymphocyte ratio as covariates to examine the association between the Supra‐Blan_2_t score (sugar, platelet‐lymphocyte ratio, age, bilirubin, AF, NIHSS, neutrophil, and TnI) and poor 3‐month outcome. This analysis was also performed in the independent validation cohort to assess the external validity of the score.

All statistical analyses were conducted using the SPSS Statistic 25.0 and MedCalc 15.2.0 software. All significance testing was two sided, and the *p* < 0.05 indicated a statistically significant difference.

## 3 RESULTS

### 3.1 Characteristics of derivation and validation cohorts

Of the 433 patients in the derivation cohort, 293 (67.7%) were men, and the mean age (SD) was 65.25 (12.50) years. Of the 525 patients in the validation cohort, 369 (70.3%) were male, and the mean age (SD) was 66.57 (11.95) years. Different clinical characteristics of, and the comparison between, the derivation and validation cohorts were presented in Table 1.

**TABLE 1:** Characteristics of, and the comparison between, the derivation and validation cohorts.

	Derivation cohort (*n* = 433)	Validation cohort (*n* = 525)	*p* Value
Demographic characteristics			
Age, years	65.25 ± 12.50	66.57 ± 11.95	0.012
Male, *n* (%)	293 (67.7)	369 (70.3)	0.362
Medical history			
History of ischemic stroke, *n* (%)	53 (12.2)	116 (22.1)	<0.001
History of intracerebral hemorrhage, *n* (%)	5 (1.2)	17 (3.2)	0.076
History of AF, *n* (%)	51 (11.8)	72 (13.7)	0.205
History of hypertension, *n* (%)	283 (65.4)	350 (66.7)	0.800
History of hyperlipemia, *n* (%)	68 (15.7)	62 (11.8)	0.048
History of diabetes mellitus, *n* (%)	98 (22.6)	124 (23.6)	0.915
Current cigarette smoking, *n* (%)	146 (33.7)	184 (35.0)	0.983
Current alcohol drinking, *n* (%)	108 (24.9)	92 (17.5)	0.003
Admission characteristics			
Baseline NIHSS score	4.00 (2.00–8.00)	5.00 (3.00–10.00)	<0.001
Baseline SBP, mmHg	146.25 ± 22.25	151.86 ± 51.11	0.024
Baseline DBP, mmHg	84.26 ± 14.84	85.96 ± 14.30	0.027
OTT, min	214.50 (150.00–263.75)	150.00 (105.00–194.00)	<0.001
Infarct volume, mm^3^	8198.51 (0–30399.52)	17734.80 (0–99024.85)	0.053
Admission laboratory values			
Baseline glucose, mmol/L	6.71 (5.44–8.70)	6.91 (5.67–9.53)	0.440
Baseline leukocyte count, g/L	6.73 (5.46–8.57)	7.07 (5.64–9.00)	0.187
Baseline neutrophil count, g/L	4.90 (3.60–6.80)	4.78 (3.55–6.64)	0.251
Baseline lymphocyte count, g/L	1.34 (0.94–1.72)	1.45 (0.97–2.10)	0.014
Baseline platelet count, g/L	190.00 (159.00–232.00)	199.11 (157.81–236.00)	0.882
Baseline D‐dimer, mg/L FEU	0.39 (0.27–0.74)	0.79 (0.01–0.88)	<0.001
Baseline FIB, g/L	3.25 (2.76–3.73)	3.10 (2.64–4.09)	0.162
Baseline INR	1.02 (0.97–1.08)	1.01 (0.93–1.13)	0.446
Baseline APTT, s	35.10 (32.33–38.10)	30.90 (27.79–34.40)	<0.001
Baseline PT, s	13.20 (12.70–13.80)	11.70 (10.70–12.70)	<0.001
Baseline total bilirubin, μmol/L	10.05 (7.40–13.88)	11.60 (9.05–14.30)	0.003
Baseline direct bilirubin, μmol/L	3.90 (3.00–5.50)	3.00 (1.91–4.41)	<0.001
Baseline indirect bilirubin, μmol/L	6.20 (4.20–8.70)	8.30 (5.99–10.70)	<0.001
TnI, ng/L	4.05 (2.30–9.55)	9.30 (25.90–599.98)	<0.001
Outcome			
mRS at 3 months			
0–2	328	356	0.001
3–6	105	169	

Abbreviations: AF, atrial fibrillation; APTT, partial thromboplastin time; DBP, diastolic blood pressure; FIB, fibrinogen; INR, international normalized ratio; mRS, modified Rankin Scale; NIHSS, National Institutes of Health Stroke Scale score; OTT, onset‐time to treatment; PT, prothrombin time; SBP, systolic blood pressure; TnI, Troponin I.

### 3.2 Univariate and multivariate analyses

As presented in Table 2, 10 potential covariates for poor 3‐month functional outcome in the derivation cohort were identified via exploratory univariate logistic regression analysis (all *p* < 0.05). The final multivariate model included eight of these variables in the derivation cohort: age >70 years, history of AF, admission NIHSS score, admission blood sugar level >6 mmol/L, admission neutrophil count >6 g/L, admission direct bilirubin level >5 μmol/L, admission TnI level >26 ng/L, and platelet–lymphocyte ratio >180.

**TABLE 2:** Derivation cohort: Univariate and multivariate logistic regression analysis of variables predicting poor 3‐month functional outcome.

	Univariate			Multivariate		
	ORs	95% CIs	*p* Value	ORs	95% CIs	*p* Value
Age, years						
≤70	1			1		
>70	2.314	1.562–3.428	<0.001	1.789	1.003–3.191	0.023
History of AF						
No	1			1		
Yes	4.962	2.887–8.529	<0.001	2.354	1.111–4.992	0.026
History of hypertension						
No	1			1		
Yes	1.504	1.012–2.235	0.044	1.383	0.258–7.400	0.705
Current cigarette smoking						
No	1			1		
Yes	0.684	0.458–1.022	0.064	0.952	0.380–2.380	0.915
Baseline NIHSS score						
0–7	1		–	1		–
8–15	12.663	7.798–20.564	<0.001	8.617	4.697–5.807	<0.001
≥16	18.01	9.149–35.455	<0.001	8.52	3.726–19.487	<0.001
Baseline SBP, mmHg						
No	1			1		
Yes	1.008	0.999–1.017	0.067	1.009	0.995–1.024	0.211
Infarct volume, mm^3^						
≤9200	1			1		
>9200	4.265	2.485–7.318	<0.001	1.349	0.631–2.882	0.44
OTT, min						
<180	1			1		
180–270	1.101	0.733–1.653	0.642	2.062	0.926–4.591	0.077
>270	0.886	0.522–0.505	0.655	2.282	0.727–0.232	0.585
Baseline glucose, mmol/L						
≤6	1			1		
>6	1.83	1.210–2.770	0.004	2.235	1.231–4.057	0.008
Baseline neutrophil count, g/L						
≤6	1			1		
>6	1.635	1.124–2.378	0.01	3.532	1.500–8.316	0.004
Baseline direct bilirubin, μmol/L						
≤5	1			1		
>5	1.549	1.053–2.279	0.001	1.278	1.016–1.608	0.036
Baseline TnI, ng/L						
≤26	1			1		
>26	3.225	1.880–5.535	<0.001	2.747	1.071–7.045	0.035
Platelet‐lymphocyte ratio						
≤180	1			1		
>180	1.705	1.162–2.501	0.006	1.973	1.909–4.284	0.035

Abbreviations: AF, atrial fibrillation; NIHSS, National Institutes of Health Stroke Scale score; OTT, onset‐time to treatment; SBP, systolic blood pressure; TnI, Troponin I.

### 3.3 Construction of the Supra‐Blan_2_t risk score

An 11‐point Supra‐Blan_2_t score was created based on three clinical variables (age >70 years, history of AF, and admission NIHSS score) and five multisystem parameters (admission blood sugar level >6 mmol/L, admission neutrophil count >6 g/L, admission direct bilirubin level >5 μmol/L, admission TnI level >26 ng/L, and platelet‐lymphocyte ratio >180) after the final multivariate logistic analysis performed before IVT therapy. Each of the eight predictor variables in the scoring system was weighted, and points were assigned based on the strength of association with corresponding *β* coefficients (Table 3).

**TABLE 3:** Supra‐Blan_2_t score: Point assignment based on the regression *β* coefficients in the derivation cohort.

Category	Points
Age, years	
≤70	0
>70	1
History of AF	
No	0
Yes	1
Baseline NIHSS score	
0–7	0
8–15	2
≥16	3
Baseline glucose, mmol/L	
≤6	0
>6	1
Baseline neutrophil count, g/L	
≤6	0
>6	2
Baseline direct bilirubin, μmol/L	
≤5	0
>5	1
Baseline TnI, ng/L	
≤26	0
>26	1
Platelet–lymphocyte ratio	
≤180	0
>180	1

Abbreviations: AF, atrial fibrillation; NIHSS, National Institutes of Health Stroke Scale score; TnI, Troponin I.

### 3.4 Performance of the risk score in the derivation cohort

The Supra‐Blan_2_t score showed good discrimination and calibration in the derivation cohort (C statistic, 0.821; *p* value of the Hosmer–Lemeshow test, 0.416) for predicting poor 3‐month functional outcome. The diagnostic accuracy in terms of sensitivity, specificity, PPVs, NPVs, and likelihood ratios is depicted in Table 4. Further, the risk of poor 3‐month outcome increased with an increase in the Supra‐Blan_2_t score (Table S1; *p <* 0.001 for trend). Furthermore, after adjusting for age, history of AF, admission NIHSS score, admission blood sugar level, admission neutrophil count, admission direct bilirubin level, admission TnI level, and platelet–lymphocyte ratio, a 1‐point increase in the Supra‐Blan_2_t score was found to be associated with poor 3‐month functional outcome based on the multivariable logistic regression analysis (adjusted ORs, 1.443 [95% CIs, 1.180–1.765], *p* < 0.001).

**TABLE 4:** Derivation cohort: Diagnostic accuracy of the Supra‐Blan_2_t score for predicting poor 3‐month functional outcome.

Score	Proportion of patients (%)	Sensitivity (95% CIs)	Specificity (95% CIs)	PPVs (95% CIs)	NPVs (95% CIs)	PLRs (95% CIs)	NLRs (95% CIs)
≥0	100	24.25 (20.34–28.62)	NA	100.00 (95.60–100.00)	0 (0–1.44)	NA	NA
≥2	94.3	27.65 (23.08–32.60)	92.00 (83.40–97.01)	94.29 (88.27–97.31)	21.04 (19.54–22.61)	3.46 (1.58–7.58)	0.79 (0.72–0.86)
≥4	75.2	36.07 (29.71–42.82)	87.85 (82.71–91.91)	75.24 (67.05–81.94)	57.32 (54.58–60.01)	2.97 (1.99–4.43)	0.73 (0.65–0.81)
≥6	57.1	58.82 (48.64–68.48)	86.40 (82.23–89.91)	57.14 (49.28–64.66)	87.20 (84.32–89.61)	4.33 (3.15–5.94)	0.48 (0.38–0.60)
≥8	23.8	73.53 (55.64–87.12)	79.95 (75.68–83.77)	23.81 (19.09–29.28)	97.26 (95.28–98.42)	3.67 (2.77–4.86)	0.33 (0.19–0.58)
≥10	3.8	100.00 (39.76–100.00)	76.46 (72.15–80.39)	3.81 (1.23–10.03)	100.00 (98.56–100.00)	4.25 (3.58–5.04)	NA

Abbreviations: NA, not available; NLRs, negative likelihood ratios; NPVs, negative predictive values; PLRs: positive likelihood ratios; PPVs, positive predictive values.

**FIGURE 2:**
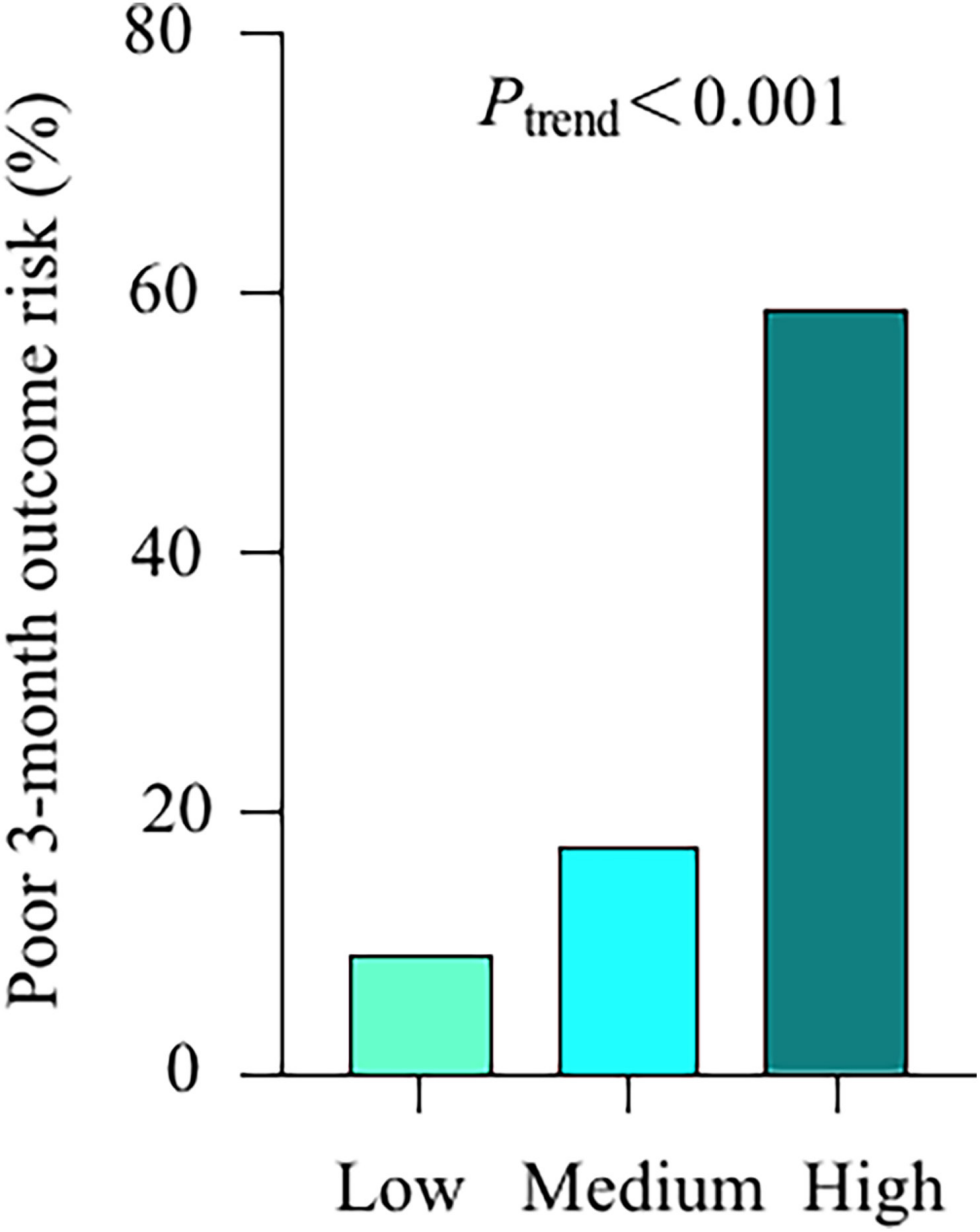
Derivation cohort: risk of poor 3‐month functional outcome in low, medium, and high Supra‐Blan_2_t categories.

When the score was stratified into low‐risk (score, 0–2), medium‐risk (score, 3–5), and high‐risk (score, ≥6) categories, a linear increase in poor 3‐month functional outcome was observed (*p* < 0.001 for trend), with poor 3‐month functional outcome risk of 9.2% (14/153 patients) in the low‐risk category, 17.4% (31/178 patients) in the medium‐risk category, and 58.8% (60/102 patients) in the high‐risk category (Table 5 and Figure 2).

**TABLE 5:** Risk of poor 3‐month outcome stratified by Supra‐Blan_2_t risk categories (Low, 0–2; Medium, 3–5; and High, ≥6) in derivation cohort.

Supra‐Blan_2_t risk categories	Poor 3‐month outcome (No. of patients)	Total (No. of patients)	Risk, %	*p* (trend)
Low (0–2)	14	153	9.2	<0.001
Medium (3–5)	31	178	17.4	
High (≥6)	60	102	58.8	
Total	105	433	–	

### 3.5 Performance of the risk score in the validation cohort

Similar to the results obtained in the derivation cohort, the Supra‐Blan_2_t score also showed good discrimination and calibration in the validation cohort (C statistic, 0.843; *p* value of the Hosmer–Lemeshow test, 0.193) for predicting poor 3‐month functional outcome. The diagnostic accuracy in terms of sensitivity, specificity, PPVs, NPVs, and likelihood ratios is presented in Table S2. Moreover, the risk of poor 3‐month outcome increased with increasing Supra‐Blan_2_t score (Table S3; *p* < 0.001 for trend). Further, after adjusting for age, history of AF, admission NIHSS score, admission blood sugar level, admission neutrophil count, admission direct bilirubin level, admission TnI level, and platelet–lymphocyte ratio, a 1‐point increase in the Supra‐Blan_2_t score was found to be associated with poor 3‐month functional outcome based on the multivariable logistic regression analysis (adjusted ORs, 1.559 [95% CIs, 1.224–1.986], *p* < 0.001).

When the score was stratified into low‐risk (score, 0–2), medium‐risk (score, 3–5), and high‐risk (score, ≥6) categories, a linear increase in poor 3‐month functional outcome was observed (*p* < 0.001 for trend), with poor 3‐month functional outcome risk of 12.7% (27/212 patients) in the low‐risk category, 36.5% (88/241 patients) in the medium‐risk category, and 73.6% (53/72 patients) in the high‐risk category (Table S4 and Figure S1). The ORs for the study outcome across increasing categories of the Supra‐Blan_2_t score indicated that a robust capacity for risk stratification was in the same order of magnitude in both the derivation and validation cohorts (e.g., 20.085 and 18.083, respectively, for high‐risk [score, ≥6] vs. low‐risk [score, 0–2] category; Table S5).

### 3.6 Receiver‐operating characteristic analysis comparing the Supra‐Blan_2_t score with other predictive scores

**FIGURE 3:**
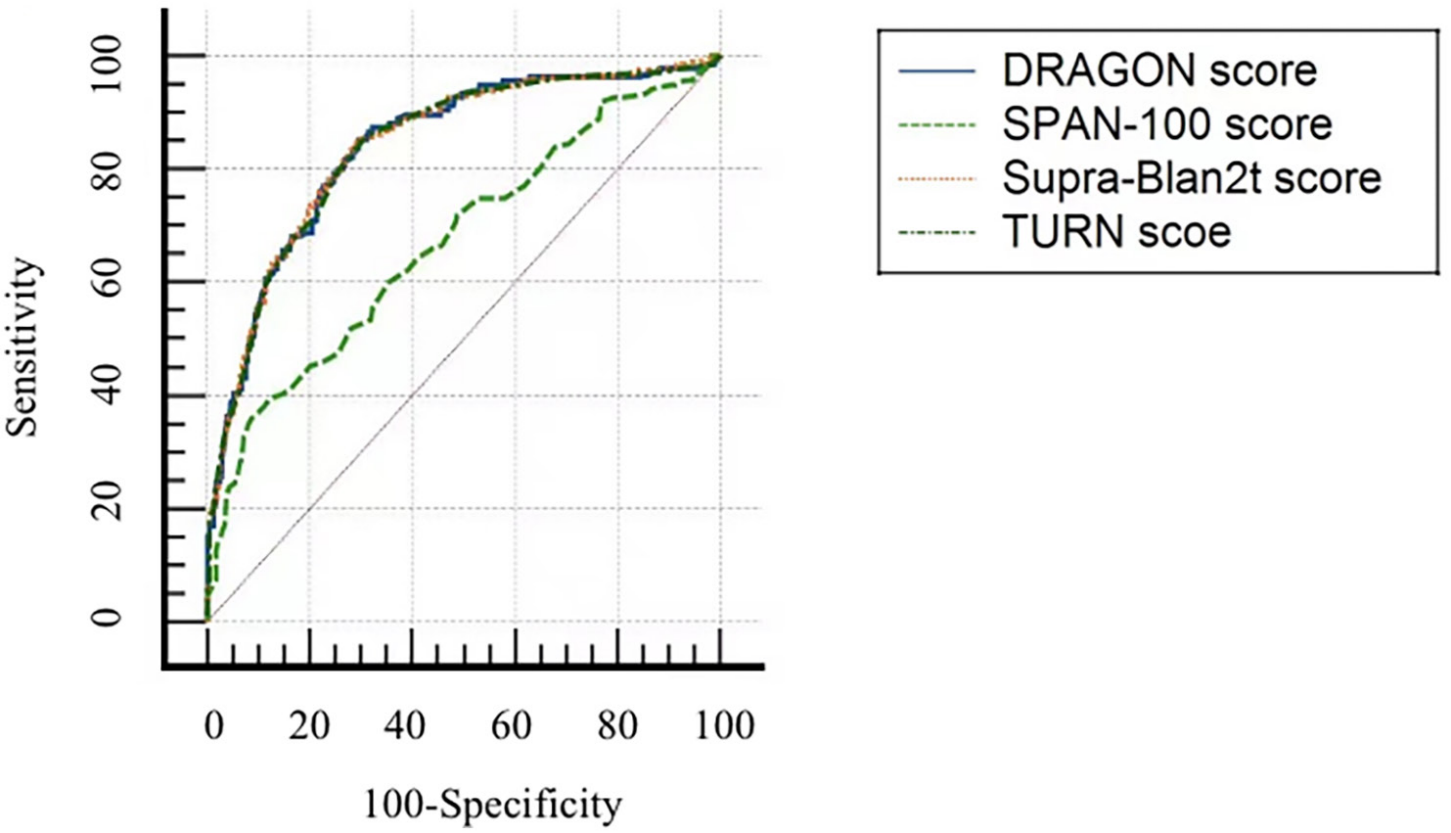
Receiver operator characteristic curve analysis comparing Supra‐Blan_2_t score with DRAGON, SPAN‐100, and TURN scores.

The AUC of the ROC curve for the Supra‐Blan_2_t score was similar to or superior to the DRAGON, TURN, and SPAN‐100 scores in predicting poor 3‐month functional outcome. The AUC value was 0.843 for the Supra‐Blan_2_t score, but 0.839 for the DRAGON score (*p* = 0.584), 0.833 for the TURN score (*p* = 0.833), and 0.697 for the SPAN‐100 score (*p* < 0.001; Table 6 and Figure 3).

**TABLE 6:** Comparison of area under the curves (AUCs) among Supra‐Blan_2_t and other predictive scores for poor functional outcome at 3 months.

	AUC (95% CIs)	*p* Value
Supra‐Blan_2_t	0.843 (0.829–0.857)	–
DRAGON	0.839 (0.823–0.855)	0.584
TURN	0.842 (0.829–0.855)	0.833
SPAN‐100	0.697 (0.680–0.713)	<0.001

## 4 DISCUSSION

Given that not all patients with AIS responded well to the IVT therapy due to the complex pathophysiological mechanisms involving multiple remote organs beyond the ischemic brain, a multisystem‐based risk score predicting the prognosis of thrombolysis in patients simply and effectively needed to be urgently developed. Hence, we proposed the Supra‐Blan_2_t score in the TRAIS study, which reliably assessed the 3‐month functional outcome in patients with acute stroke treated with IVT to facilitate treatment decisions and communicate prognostic predictions to patients and their families. The Supra‐Blan_2_t score integrated eight easily accessible baseline variables, including admission blood sugar, platelet‐lymphocyte ratio, age, direct bilirubin, history of AF, admission NIHSS score, admission neutrophil count, and TnI level, with a total score ranging from 0 to 11. Importantly, the score included multisystem indicators to reflect the damage to remote peripheral organs besides primary ischemia brain injury, presenting excellent discriminative and calibrated characteristics in the derivation cohort, which was further confirmed in the validation cohort. Moreover, the heterogeneity observed between the derivation and validation cohorts supported the generalization of the score.

**FIGURE 4:**
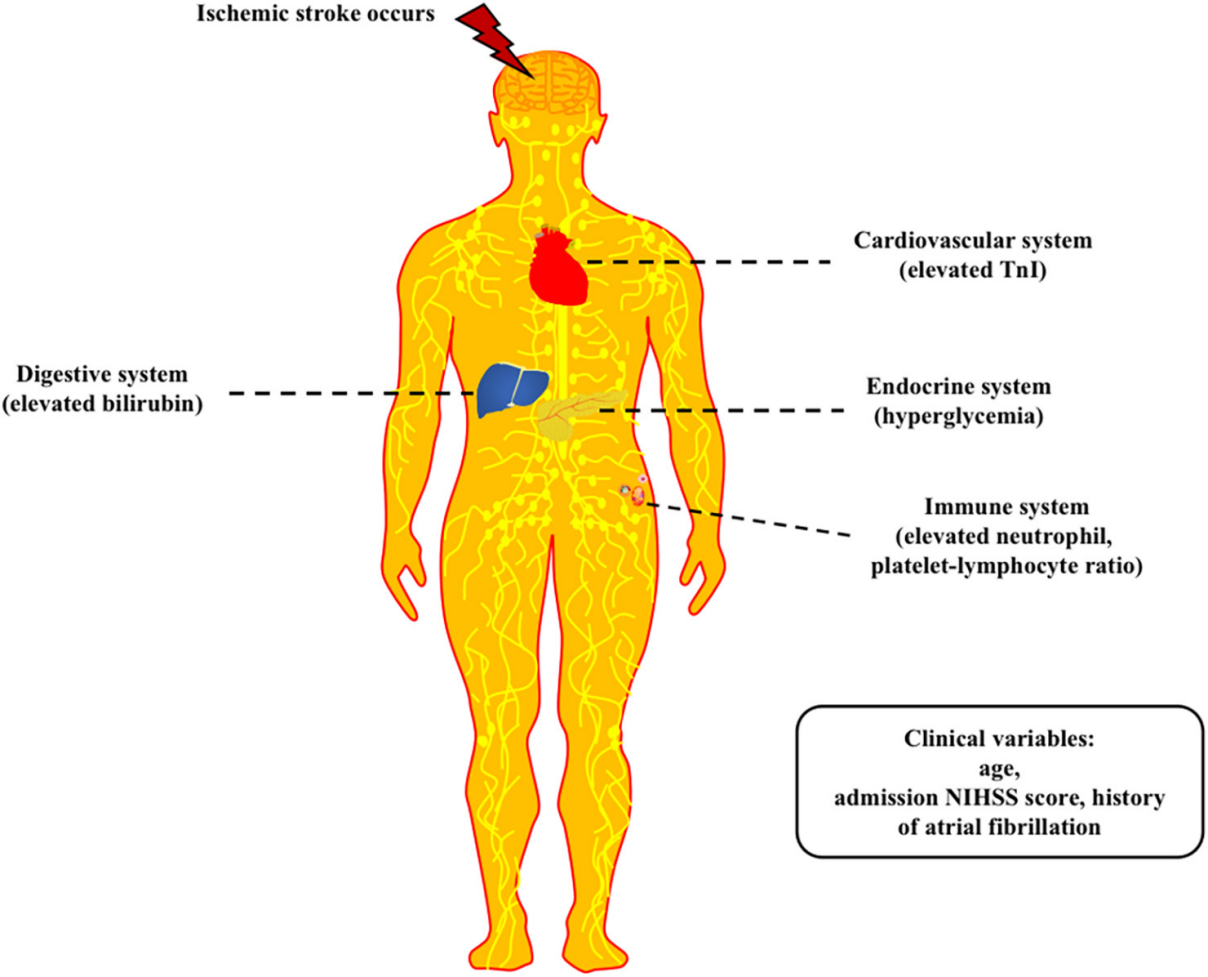
Multisystem parameters and clinical variables involvement in Supra‐Blan_2_t prediction score. Multisystem included cardiovascular system, endocrine system, immune system, and digestive system, while clinical variables included age, admission National Institutes of Health Stroke Scale (NIHSS) score, and history of atrial fibrillation.

Shortly after the stroke, peripheral immunosuppression accompanied by the overstimulation of autonomic and neuroendocrine pathways, and the injury to the motor pathway could lead to the dysfunction of respiratory, urinary, cardiovascular, gastrointestinal, musculoskeletal, and endocrine systems.^4^, ^15^ Previous studies demonstrated that the hyperglycemic state after stroke was a key factor in antagonizing the therapeutic effect of thrombolytic therapy, which could be attributed to the mechanisms of impaired vessel recanalization, increased ischemia–reperfusion injury, and the blood–brain barrier disruption under hyperglycemic state.^16^, ^17^ In this study, elevated serum TnI levels reflecting cardiac dysfunction and a history of AF were found to be associated with poor clinical outcomes, which was in line with previous findings.^18^, ^19^ In recent years, the significant role of inflammation in the pathophysiological of stroke has been increasingly recognized,^20^ in which both neutrophil count and platelet–lymphocyte ratio were considered as novel biomarkers of the severity of systemic inflammation, resulting in poor functional outcomes in patients with AIS receiving IVT therapy.^21^, ^22^ In particular, evidence suggested the role of inflammation in the pathophysiologic formation of stroke thrombus,^23^, ^24^ in which both neutrophil count and platelet–lymphocyte ratio revealed a thrombotic pathway. Direct bilirubin, previously demonstrated to be a biomarker for outcomes in patients in the TRAIS cohort of this study,^25^ was also included in our prediction score. Finally, the traditional prognostic prediction variables significantly associated with outcomes such as age and the admission NIHSS score were also included.^7^ Hence, the Supra‐Blan_2_t score comprised traditional prognostic prediction variables in combination with multisystem injury markers (Figure 4).

In both derivation and validation cohorts, the Supra‐Blan_2_t score was stratified into three risk categories, indicating that the risk of poor 3‐month functional outcome increased stepwise across increasing Supra‐Blan_2_t score categories. A prediction scale for estimating the risk of poor 3‐month functional outcome after IVT therapy may be clinically implemented based on two non‐mutually exclusive approaches. On the one hand, patients at low risk (score, 0–2) would benefit from less close clinical monitoring, thereby saving medical costs. On the other hand, when the predicted risk of poor 3‐month functional outcome is elevated among medium‐ and high‐risk patients, a multifaceted intervention should be applied based on closer follow‐up monitoring in such patients to increase the possibility of obtaining good functional recovery after 3 months.

Different prediction scores have been developed for 3‐month clinical prognostication in patients with AIS receiving IVT therapy. However, the defects of these models in a clinical application cannot be ignored. For example, Strbian et al. created the DRAGON score composed of the imaging parameter of CT head scan, prestroke mRS score, age, admission blood sugar level, onset‐time to treatment, and admission NIHSS score to validate its predictive power in a validation cohort of 333 patients.^9^ In the DRAGON score, the radiological recognition of (hyper) dense cerebral artery sign or early infarct sign upon admission using brain CT was subtle and complex owing to the huge subjectivity and limited imaging experience of neurologists,^26^, ^27^ making it difficult to implement in grassroots medical and health service systems in China. Asuzu et al.^28^ calculated the TURN score for each patient in a multicenter cohort as follows: TURN = −4.65 + (prestroke mRS × 0.27) + (admission NIHSS score × 0.10). However, no details were provided on the performance of the score, and hence, we could not externally validate the discriminative power of the aforementioned equation. Saposnik et al.^7^ created the SPAN‐100 score. Nevertheless, this score involved too few variables and lacked external validation, leading to diagnostic inaccuracy.

Compared with the aforementioned prediction scores, the Supra‐Blan_2_t score exhibited several advantages. First, we enrolled consecutive patients from both large hospitals and primary care providers based on a large‐sample multicenter TRAIS study. Second, new independent predictors comprehensively reflecting the stress state of the body and widely available in routine practice with short turnaround times and no dedicated laboratory equipment, including platelet–lymphocyte ratio, direct bilirubin, neutrophil, and TnI, were added to the risk prediction model. Third, we validated the discriminative power and diagnostic accuracy of the score in an independent cohort of 525 patients recruited from 13 centers in the Hubei province. Hence, the Supra‐Blan_2_t score was found to be most feasible and applicable in clinical settings in China compared with the DRAGON score, TURN score, and SPAN‐100 index.

This study also had several limitations. First, all participating hospitals in this TRAIS study were urban hospitals with more resources and specialists than hospitals in rural areas. Therefore, the study might have a selection bias. Second, although we controlled known stroke outcome predictors, we could not rule out the possibility that additional baseline variables (unmeasured confounding factors) might also have some impact. Third, detailed neuroimaging information on the status of vessel occlusion, infarct core, and salvageable tissue was not considered in this score, as these data are not routinely collected for patients with acute stroke and that such imaging techniques may not be widely available. Fourth, the laboratory variables were drawn only once upon admission, and the dynamic changes of these indicators were not observed. Finally, this is a retrospective study, and an external cohort should be further stated or developed.

## 5 CONCLUSIONS

We developed and validated a novel clinical Supra‐Blan_2_t prediction score to predict poor 3‐month functional outcome. The Supra‐Blan_2_t score was created based on easily available multisystem laboratory (blood sugar level, neutrophil count, direct bilirubin level, platelet–lymphocyte ratio, and TnI level) and clinical variables (age, admission NIHSS score, and history of AF) in patients with acute stroke treated with IVT therapy, featuring good calibration and discrimination. And the results of this study should be understood in the context of its retrospective design.

## AUTHOR CONTRIBUTIONS

Huijuan Jin, Qiwei Peng, and Min Li performed major research design and wrote the article; Shuai Sun, Jinghua Zhou, and Jichuan Hu analyzed data and edited the article; Ming Huang, Xinglong Chen, and Yanan Li assessed the functional outcome of patients; Yifan Zhou, Yan Wan, Candong Hong, and Shengcai Chen interpreted results; Bo Hu conceived the study and analyzed data; and all authors reviewed the article.

## FUNDING INFORMATION

This work was supported by the National Natural Science Foundation of China (Grants: 82090044 and 81820108010 to Bo Hu; 82171306 to Huijuan Jin), and the National Key Research and Development Program of China (2018YFC1312200 to Bo Hu).

## CONFLICT OF INTEREST STATEMENT

The authors declare that they have no competing interests. Hu Bo is an Editorial Board member of CNS Neuroscience and Therapeutics and a coauthor of this article. To minimize bias, they were excluded from all editorial decision‐making related to the acceptance of this article for publication.

## Supporting information


Figure S1.

Tables S1–S5.

## Data Availability

The data that support the findings of this study are available from the corresponding author upon reasonable request.
