# Supplementary figures and images for: Supra‐Blan2t score as a multisystem‐based risk score to predict poor 3‐month outcome in acute ischemic stroke patients with intravenous thrombolysis
Source: CNS Neurosci Ther. 2023 Jul 30;30(2):e14381. doi: 10.1111/cns.14381 (PMC10848105; doi:10.1111/cns.14381)

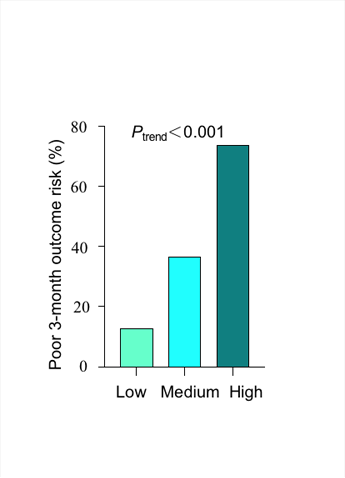

Supplement: Supplementary file 1 — Figure S1. [file CNS-30-e14381-s002.tif]
