# Supplementary material for: Supra‐Blan2t score as a multisystem‐based risk score to predict poor 3‐month outcome in acute ischemic stroke patients with intravenous thrombolysis
Source: CNS Neurosci Ther. 2023 Jul 30;30(2):e14381. doi: 10.1111/cns.14381 (PMC10848105; doi:10.1111/cns.14381)

**Supplementary Online Content**

**Table S1 Risk of poor 3-month outcome stratified by Supra-Blan_2_t score in derivation cohort**

**Table S2 Validation cohort: diagnostic accuracy of the Supra-Blan_2_t score for predicting poor 3-month functional outcome**

**Table S3 Risk of poor 3-month outcome stratified by Supra-Blan_2_t score in validation cohort**

**Table S4 Risk of poor 3-month outcome stratified by Supra-Blan_2_t risk categories (Low=0-2; Medium=3-5; and High≥6) in validation cohort**

**Table S5 ORs across Supra-Blan_2_t score risk categories (High-, Medium- and Low-risk groups) for poor 3-month functional outcome in the derivation and validation cohorts.**

**Figure S1 Validation cohort: risk of poor 3-month functional outcome in low, medium and high Supra-Blan_2_t categories**

| **Table S1 Risk of poor 3-month outcome stratified by Supra-Blan_2_t score in derivation cohort** | | | | |
| --- | --- | --- | --- | --- |
| Supra-Blan_2_t score | poor 3-month outcome (NO. patients) | Total (NO. patients) | Risk, % | *P* (trend) |
| 0 | 3 | 41 | 7.3 | ＜0.001 |
| 1 | 3 | 34 | 8.8 |  |
| 2 | 8 | 78 | 10.3 |  |
| 3 | 12 | 61 | 19.7 |  |
| 4 | 8 | 64 | 12.5 |  |
| 5 | 11 | 53 | 20.8 |  |
| 6 | 16 | 36 | 44.4 |  |
| 7 | 19 | 32 | 59.4 |  |
| 8 | 14 | 20 | 70.0 |  |
| 9 | 7 | 10 | 70.0 |  |
| ≥10 | 4 | 4 | 100.0 |  |
| Total | 105 | 433 | - |  |

| **Table S2 Validation cohort: diagnostic accuracy of the Supra-Blan_2_t score for predicting poor 3-month functional outcome** | | | | | | | |
| --- | --- | --- | --- | --- | --- | --- | --- |
| Score | Proportion of patients (%) | Sensitivity (95% CIs) | Specificity (95% CIs) | PPVs (95% CIs) | NPVs (95% CIs) | PLRs (95% CIs) | NLRs (95% CIs) |
| ≥0 | 100 | 31.54 (27.60-35.75) | NA | 100.00 (97.15-100.00) | 0 (0-1.33) | NA | NA |
| ≥2 | 94.5 | 35.88 (31.39-40.63) | 89.77 (81.01-94.93) | 94.51 (89.52-97.30) | 22.19 (18.06-26.94) | 3.51 (1.87-6.60) | 0.71 (0.66-0.77) |
| ≥4 | 69.5 | 53.77 (46.82-60.59) | 83.77 (79.06-87.61) | 69.51 (61.77-76.32) | 72.47 (67.46-76.98) | 3.31 (2.50-4.39) | 0.55 (0.48-0.64) |
| ≥6 | 34.8 | 74.03 (62.57-83.05) | 75.85 (71.53-79.70) | 34.76 (27.61-42.63) | 94.38 (91.31-96.45) | 3.06 (2.48-3.79) | 0.34 (0.24-0.50) |
| ≥8 | 4.9 | 80.00 (44.22-96.46) | 69.41 (65.18-73.35) | 4.88 (2.29-9.72) | 99.44 (97.76-99.90) | 2.62 (1.87-3.66) | 0.29 (0.08-0.99) |
| ≥10 | 0.6 | 100.00 (5.46-100.00) | 68.59 (64.38-72.53) | 0.61 (0.03-3.86) | 100.00 (98.67-100.00) | 3.18 (2.80-3.62) | NA |

Abbreviations: PPVs, positive predictive values; NPVs, negative predictive values; PLRs: positive likelihood ratios; NLRs, negative likelihood ratios; NA, not available.

| **Table S3 Risk of poor 3-month outcome stratified by Supra-Blan_2_t score in validation cohort** | | | | | | | | | |  |
| --- | --- | --- | --- | --- | --- | --- | --- | --- | --- | --- |
| Supra-Blan_2_t score | poor 3-month outcome (NO. patients) | | Total (NO. patients) | | Risk, % | | | *P* (trend) | |  |
| 0 | 1 | | 19 | | 5.3 | | | ＜0.001 | |  |
| 1 | 10 | | 81 | | 12.3 | | |  |  |  |
| 2 | 16 | | 112 | | 14.3 | | |  |  |  |
| 3 | 25 | | 107 | | 23.4 | | |  |  |  |
| 4 | 32 | | 76 | | 42.1 | | |  |  |  |
| 5 | 31 | | 58 | | 53.4 | | |  |  |  |
| 6 | 23 | | 36 | | 63.9 | | |  |  |  |
| 7 | 22 | | 26 | | 84.6 | | |  |  |  |
| 8 | 5 | | 7 | | 71.4 | | |  |  |  |
| 9 | 2 | | 2 | | 100.0 | | |  |  |  |
| ≥10 | 1 | | 1 | | 100.0 | | |  |  |  |
| Total | 168 | | 525 | | - | | |  | |  |
| **Table S4 Risk of poor 3-month outcome stratified by Supra-Blan_2_t risk categories (Low=0-2; Medium=3-5; and High≥6) in validation cohort** | | | | | | | | | | |
| Supra-Blan_2_t risk categories | | poor 3-month outcome (No. patients) | | Total (No. patients) | | | Risk, % | | *P* (trend) | |
| Low (0-2) | | 27 | | 212 | | 12.7 | | | | ＜0.001 |
| Medium (3-5) | | 88 | | 241 | | 36.5 | | | |  |
| High≥6 | | 53 | | 72 | | 73.6 | | | |  |
| Total | | 168 | | 525 | | - | | | |  |

| **Table S5 ORs across Supra-Blan_2_t score risk categories (High-, Medium- and Low- risk groups) for poor 3-month functional outcome in the derivation and validation cohorts.** | | | | | |
| --- | --- | --- | --- | --- | --- |
| Supra-blan_2_t score risk categories | Derivation cohort | |  | Validation cohort | |
|  | ORs | 95% CIs |  | ORs | 95% CIs |
| poor 3-month functional outcome |  | | | | |
| High-risk (score ≥6）vs. Low-risk (score 0-2) | 20.085 | 8.455-47.715 |  | 18.083 | 9.511-34.380 |
| Medium-risk (score 3-5) vs. Low-risk (score 0-2) | 2.981 | 1.775-5.005 |  | 3.256 | 2.420-5.248 |

**Figure S1**


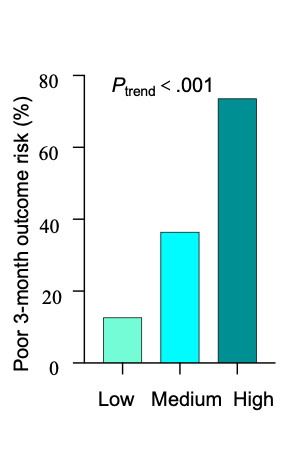

Supplement: Supplementary file 2 — Tables S1–S5. [file CNS-30-e14381-s001.docx]
